# Chitosan-capped gold nanoparticles for indicating temperature abuse in frozen stored products

**DOI:** 10.1038/s41538-019-0034-z

**Published:** 2019-01-31

**Authors:** Chitradurga Obaiah Mohan, S. Gunasekaran, C. N. Ravishankar

**Affiliations:** 1 Fish Processing Division, ICAR-Central Institute of Fisheries Technology (Indian Council of Agricultural Research), Ministry of Agriculture & Farmers Welfare, Govt. of India, Matsyapuri P.O., Cochin, 682 029 India; 2 Biological Systems Engineering Department, University of Wisconsin, Madison, WI USA; 3 ICAR-Central Institute of Fisheries Technology (Indian Council of Agricultural Research), Ministry of Agriculture & Farmers Welfare, Govt. of India, Matsyapuri P.O., Cochin, 682 029 India

**Keywords:** Biochemistry, Other nanotechnology

## Abstract

The present study aimed to optimize the conditions for the synthesis of gold nanoparticles (AuNP) using chitosan and to assess its effectiveness as temperature threshold indication for frozen storage conditions. Chitosan concentration of 0.25% and temperature of 90 °C for 15 min was optimum for synthesizing AuNP. The maximum absorbance (*λ*_max_) was observed at 530 and 540 nm for 0.125% and 0.25% chitosan, respectively, indicating shifting of peak toward longer wavelengths (red shift) with increasing chitosan concentration indicating larger AuNPs. A prominent absorption peak at 1367 cm^−1^ by Fourier transform-infrared (FTIR) spectrum corresponding to C−C stretching of the glucosamine group of chitosan indicates the chitosan capping on the AuNP. Higher peak intensity and a peak shift toward shorter wavelength were observed for AuNPs exposed to frozen temperature abused conditions. Distinctly clear visible color variation from cherry red to gray indicates its application as temperature abuse indicator in frozen products.

## Sensors: Chitosan capped gold nanoparticles indicate storage temperature history

Thermal history indicators (THI) attached on packages can tell us the storage temperature history of food and pharmaceutical products, which influences their quality and safety a lot. Metal nanoparticles are widely considered as THI. Mohan from ICAR-Central Institute of Fisheries Technology, India and coworkers reported a temperature abuse indictor based on chitosan capped gold nanoparticles. Chitosan was used as a reducing and stabilizing agent in the synthesis of gold nanoparticles. The final size of Au nanoparticles was determined by the used concentration of chitosan. The chitosan capped gold nanoparticles were capable of altering color and color intensity when exposed to temperature fluctuations. This can be attributed to the aggregation of the gold nanoparticles, alteration in shape and size at frozen temperature abused conditions.

## Introduction

Temperature is the most crucial factor affecting the quality by influencing the kinetics of physical, chemical, and microbial spoilage in perishable food commodities as well as pharmaceutical products. The storage temperature of the temperature-sensitive products like chilled, refrigerated and frozen stored food, pharmaceutical, and biological products is monitored strictly to ensure that the product remains within the accepted threshold range and to ensure conformance with product release specification. At present, the temperature of frozen products in the food-processing establishments is monitored using temperature recorders. There is no mechanism for monitoring the temperature of foods during transportation and distribution and in retail stores. A visible temperature abuse indicator will be useful for maintaining the proper storage conditions at all the stages. This can be achieved by nanotechnology by developing biosensors like thermal history indicator (THI) that provides very useful information on temperature history. THI is an important type of intelligent packaging. It consists of a simple device that can be attached to the packaging containing food or pharmaceutical products to indicate its storage temperature history and thereby quality and safety of the products. Three basic types of THIs like critical temperature indicators, partial history indicators, and full history indicators are available in the market,^1^ which are either diffusion-, enzymatic-, or polymer-based systems. Development of THIs using metal nanoparticles is gaining popularity among the research community, and among the metal nanoparticles, gold nanoparticles (AuNPs) have attracted considerable attention due to its unique therapeutic activity, optical behavior, inert, and nontoxic nature. The AuNPs can be prepared by employing supercritical fluid technology, physical, chemical, and biological methods. Biological methods for nanoparticle synthesis using microorganisms, enzymes, vitamins, sugar, and biodegradable polymers of plant or animal origin have been suggested as possible ecofriendly alternatives to chemical and physical methods.^2,3^

Using chitosan, a derivative of chitin, which is one of the most abundant natural biodegradable polysaccharide extracted from crustacean waste, for nanoparticle synthesis can be advantageous over other biological processes as it avoids the elaborate process and can be easily scaled up. Chitin is the second most abundantly available natural polymer after cellulose,^4^ which can be extracted from crab, shrimps shell waste, and the bone plates of squid and cuttlefish.^5^ It is not soluble in dilute acids, whereas chitosan, which is a deacetylated form of chitin, is soluble in dilute acids. Use of chitosan for fish preservation^4,6–8^ and for the synthesis of AuNP is studied by many researchers.^9–13^ Studies on THI using biomolecules alginate^14^ and gelatin^15^ have been reported. However, there is no report on the THI using chitosan. Capping AuNPs with various chemicals and natural compounds results in alterations in the basic properties of AuNPs. Gold nanoparticles obtained by capping with chitosan can be used as temperature abuse indicator as the AuNPs change its color and intensity of the color with fluctuation in storage temperature and its duration. This variation in color and color intensity is mainly due to the alteration in size and shape of chitosan-capped AuNPs, which can be used for the development of sensors. Although many researchers have demonstrated the synthesis of AuNPs using chitosan, there are very limited reports on the use of chitosan-capped AuNPs for sensor applications. Hence, the present study was undertaken with the objective of optimizing the process conditions for synthesizing the chitosan-capped AuNPs, to characterize and to evaluate its application as temperature abuse indicator for frozen storage conditions.

## Results and discussion

### Properties of chitosan

Chitosan can be used as a reducing agent to obtain the gold nanoparticles due to its electronegativity property. It can also act as an electrostatic stabilizer as it is a polyelectrolyte, which would render dual advantage by providing sufficient charge through the amino groups, which will aid in the subsequent attachment of the biomolecules, as well as render optimum stability and subsequently help to improve the uptake of the nanoparticles. Quality characteristics of chitosan used in the present study are summarized in Table 1. The chitosan had a slightly yellowish creamy white color. The moisture content was less than 8%, and protein and ash content were 0.92% and 1.16%, respectively. The degree of deacetylation, which is one of the important properties of the chitosan,^16^ deciding its application, was found to be 81.34%. The results of the viscosity indicated that the chitosan used in the study had low viscosity (79 cP) and the pH of chitosan solution (in acetic acid) was 3.94.

**Table 1:** Properties of chitosan (mean ± standard deviation, *n* = 5)

Attributes	Results
Appearance	Slightly yellowish
Moisture (%)	7.95 ± 0.21
Protein (%)	0.92 ± 0.13
Ash (%)	1.16 ± 0.11
Degree of deacetylation (%)	81.34 ± 1.34
Viscosity (cP)	78.96 ± 1.06
pH	3.94 ± 0.05

### Optimization of synthesis condition for chitosan-capped AuNPs

**Fig. 1:**
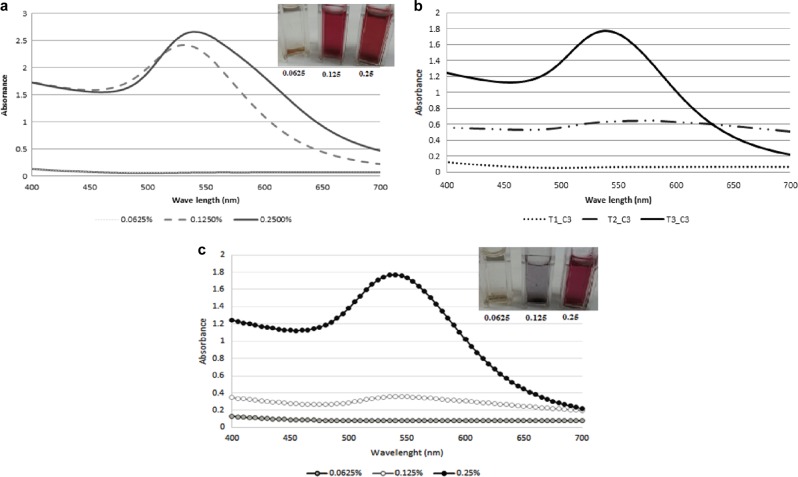
Influence of chitosan concentration (0.0625, 0.125, and 0.25%), heating time, and frozen storage on the UV−Visible spectra and visible color (inset) of chitosan-capped gold nanoparticles

Optimization of the concentration of chitosan, as well as time and temperature assumes importance as it varies with biomolecules and chemicals. For optimization of chitosan concentration, heating temperature and duration was kept constant, i.e., 90 °C for 15 min based on available literature for different biomolecules.^14,15,17^ Upon addition of HAuCl_4_^.^3H_2_O to the clear solution of chitosan, the solution turned into bright yellow color. For solutions with 0.125 and 0.25% chitosan, the color changed from bright yellow to light yellow, colorless, suggesting the reduction of Au ions into Au atoms. Upon continued heating, the solution started to turn into light pink indicating the formation of AuNPs. Further heating resulted in the formation of dark cherry red color, indicating the formation of chitosan-capped gold nanoparticles. This clearly indicates the formation of AuNPs in the concentrations of 0.125 and 0.25% chitosan. However, the color was highly intensive for the solution with 0.25% of chitosan, compared to 0.125% of chitosan, which is supported by UV-visible spectral observation. For the lower concentration of chitosan (0.0625%), the bright yellow color persisted for 10 min, which slowly changed to light yellow color by the end of 15 min of heating time. This indicates that the lower concentration of chitosan was not effective for the formation of AuNPs. The *λ*_max_ for chitosan-capped AuNPs was observed at 530 and 540 nm for 0.125% and 0.25% chitosan, respectively, and for lower chitosan concentration (0.0625%), a prominent peak was not observed (Fig. 1a). This indicates that more AuNPs were synthesized in 0.25% chitosan, compared to other concentrations at the same heating conditions, and hence this concentration was used for further optimization of heating temperature and time. UV−Visible spectra of chitosan-capped AuNPs exposed to different heating times are shown in Fig. 1b. Lower heating time of 5 and 10 min did not show any peak. Heating time of only 15 min resulted in a prominent peak indicating its suitability. Among three heating temperatures studied (70, 80, and 90 °C), higher heating temperature, i.e., 90 °C for 15 min resulted in bright cherry red-colored solution indicating the formation of chitosan-stabilized AuNPs (results not shown). The heating temperature of 80 °C resulted in bright yellow-colored solution, whereas heating at 70 °C resulted in light yellow color upon heating for 15 min. At both the lower temperatures, the solution did not turn to cherry red color, indicating that these lower heating temperatures for 15 min were not sufficient for the synthesis of AuNPs. Chitosan concentration of 0.25% (w/v), heating temperature of 90 °C for 15 min was chosen as optimal conditions for the preparation of chitosan-capped AuNPs, as indicated by the intense cherry red color. Wang et al.^15^ also reported heating temperature of 90 °C for 15 min as the optimum condition for gelatin-based AuNPs to be used as THI. The chitosan concentration was further optimized using chitosan concentrations of 0.2, 0.25, and 0.3% (w/v) and characterized as above (results not shown). Based on the spectrophotometric characteristics and visible color changes, chitosan concentration of 0.25% was found optimum.

### Characterization of chitosan-capped AuNPs

The synthesized AuNPs were characterized by UV−Visible spectroscopy, which has proven to be a very useful method for the detection of prepared metallic nanoparticles. In UV−Vis spectra, the AuNPs exhibit a localized surface plasmon resonance band at around 526 nm due to collective oscillations of the electron at the surface of the nanoparticles that is correlated with the electromagnetic field of the incoming light. In the present study, *λ*_max_ was observed at 530 and 540 nm for 0.125% and 0.25% chitosan, respectively, indicating a shift of peak to the right (red shift) (Fig. 1a). This could be due to the formation of AuNPs of various shapes, size, or concentration dependencies. In 0.0625% chitosan solution heated at 90 °C for 15 min, the peak could not be observed indicating the absence of AuNPs. The concentration of chitosan affected the size and zeta potential of AuNPs (Table 2). The size of the chitosan-capped AuNPs was 30.6 nm for 0.25% chitosan, compared to 59.8 and 175.6 nm for 0.125% and 0.0625% chitosan, respectively. The AuNPs could dissociate due to heating to form smaller particles stabilized by the amine groups on the chitosan, leading to the formation of chitosan-capped stable AuNPs. Although heating time of 15 min was maintained for all the concentrations of chitosan, reduction of Au ions into Au atoms was faster in 0.25% chitosan. The smallest particle size obtained for 0.25% chitosan solution could be due to the shorter reduction time and subsequent heating compared to other samples. Particle sizes of 7, 7−35, and 115 nm have been reported for chitosan-capped gold nanoparticles prepared using different grades of chitosan under various conditions.^12,13,18^ Polydispersity varies between 0 and 1, wherein values between 0 and 0.08 are considered as a nearly monodisperse sample, values between 0.08 and 0.7 are mid-range values, and 0.7–1.0 indicate a very broad distribution of particle size. In the case of nanoparticles, polydispersity below 0.3 is desirable and values more than 0.3 indicate polydispersity. In the present study, polydispersity varied between 0.209 and 0.247 among the different concentrations of chitosan AuNPs that indicates good dispersion of the nanoparticles. The zeta potential is the measure of surface charge of nanoparticles and it indicates the stability of the nanoparticles in the suspension system. The zeta potential was the least for 0.25% chitosan-capped AuNPs (51 mV) and increased with the decrease in the chitosan concentration (58.5 and 64.2 mV for 0.125% and 0.0625% chitosan-capped AuNPs, respectively). Zeta potential observed in the present study is similar to the one reported for gold nanoparticles prepared using 0.1% chitosan solution.^13^

**Table 2:** Particle size and zeta potential of chitosan gold nanoparticles (mean ± standard deviation, *n* = 10)

Parameters	Chitosan (0.0625%)	Chitosan (0.125%)	Chitosan (0.25%)
Effective diameter (nm)	175.6 ± 1.28^a^	59.8 ± 1.49^b^	30.6 ± 1.17^c^
Polydispersity	0.233 ± 0.01^a^	0.209 ± 0.01^b^	0.247 ± 0.02^a^
Zeta potential (mV)	64.24 ± 2.78^b^	58.58 ± 3.08^a^	51.0 ± 2.18^a^

^a,b,c^Mean values in the same row with different letters are significantly different (*P* < 0.05)

**Fig. 2:**
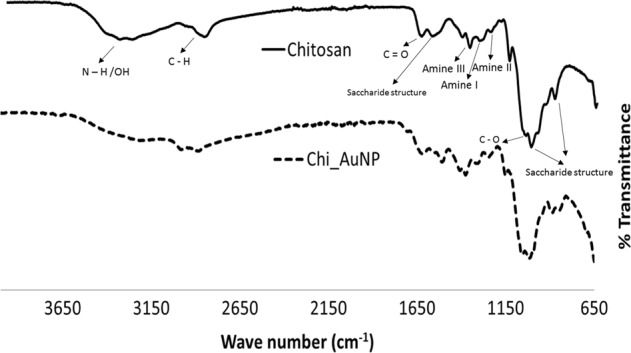
Fourier transform-infrared spectra of pure chitosan (straight line) and chitosan-capped AuNP (dashed line)

To characterize the chitosan-capped AuNPs, the functional groups and the corresponding absorption peaks of pure chitosan and chitosan-capped AuNPs were characterized using FTIR spectra (Fig. 2). FTIR spectra of pure chitosan helped in characterizing the starting material fully and it showed bands at 3357, 3271, 2856, 1639, 1579, and 1367 cm^−1^. The spectrum of pure chitosan exhibited a broad band between 3357 and 3271 cm^−1^due to the stretching vibrations of O−H or N−H groups. The peak at 2856 cm^−1^ is attributed to stretching of C−H groups. The band located at 1639 cm^−1^ is related to the vibrations of carbonyl bonds (C=O) (amide I) of O=C-NHR, while the absorption peak at 1579 cm^−1^ is related to the bending of N−H bonds (amide II) (NH_2_).^19^ The band at 1408 cm^−1^ is due to CH_3_ wagging.^20^ A prominent absorption peak at 1367 cm^−1^ could be attributed to the C−C stretching of the glucosamine group of chitosan. The absorption peak at 1016 cm^−1^ is assigned to C−O stretching. The characteristic features of the chitosan spectrum observed in the present study are similar to previous reports.^21,22^ FTIR spectra of chitosan-capped gold nanoparticles were assessed to identify the possible interactions between the functional groups of chitosan with gold nanoparticles. When two or more substances are mixed, chemical interactions are reflected by shift or change in the characteristic spectra peaks.^22^ FTIR spectra of chitosan-capped gold nanoparticles in the present study exhibited almost similar peaks as that of pure chitosan, indicating uniform deposition of chitosan over gold nanoparticles. The only difference observed in the FTIR of chitosan-capped AuNP over pure chitosan is the shift of a broad band observed between 3357–3271 cm^−1^ and 2992–2901 cm^−1^ and the disappearance of a peak observed at 2856 cm^−1^. Gold nanoparticles prepared in the present study were stored under frozen conditions to assess the stability in terms of its effective diameter. A slight increase in the size of gold nanoparticles was observed with the storage period for all the three chitosan concentrations (Table 3). However, the increase in the diameter of AuNP with the storage period was not significantly different (*P* < 0.05). The results indicated that storage under constant frozen condition has little influence on the properties of synthesized AuNPs up to 29 days.

**Table 3:** Stability of chitosan-capped gold nanoparticles stored under frozen condition

Time (days)	Effective diameter (nm)*		
	Chitosan (0.0625%)	Chitosan (0.125%)	Chitosan (0.25%)
0	175.6 ± 1.28^aA^	59.8 ± 1.49^aB^	30.6 ± 1.17^aC^
7	176 ± 2.04^aA^	62.4 ± 1.18^aB^	30.9 ± 1.82^aC^
15	178.4 ± 1.67^aA^	61.27 ± 2.04^aB^	31.87 ± 1.08^aC^
21	173.86 ± 2.18^bA^	67.54 ± 1.56^bB^	33.76 ± 1.77^bC^
29	180.91 ± 2.44^cA^	60.42 ± 1.92^aB^	34.19 ± 1.88 ^bC^

*Mean ± standard deviation, *n* = 10

^A,B,C^Mean values in the same row with different letters are significantly different (*P* < 0.05)

^a,b,c^Mean values in the same column with different letters are significantly different (*P* < 0.05)

### Evaluation of chitosan-capped AuNP as THI

**Fig. 3:**
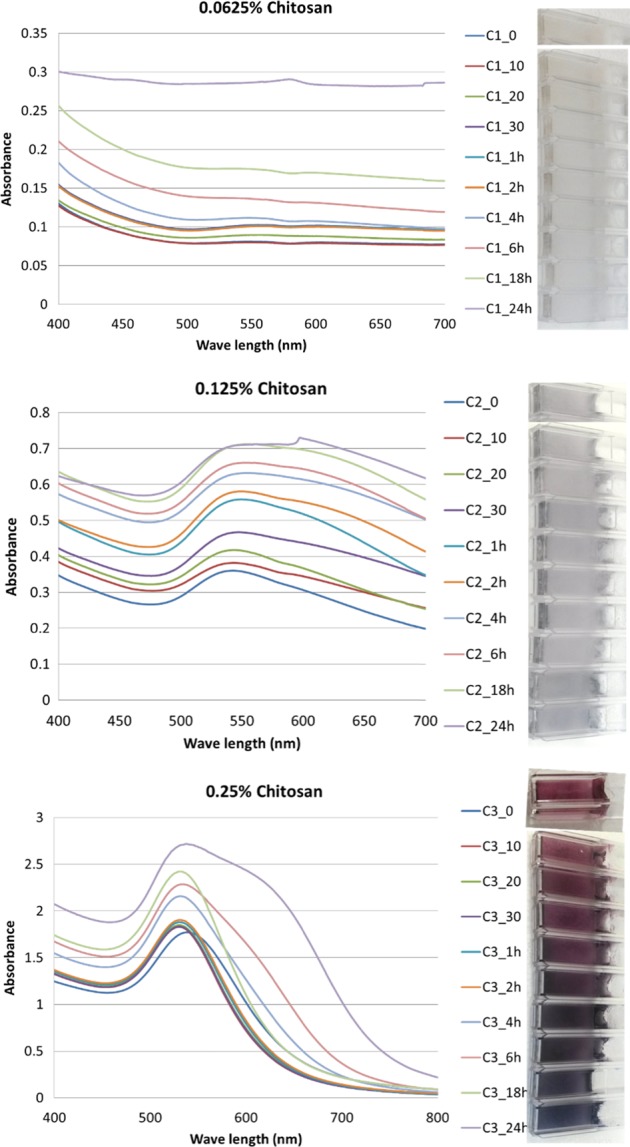
Effect of temperature abuse on the UV−Visible absorption spectra and the corresponding visible color (inset) of chitosan AuNP at different concentrations (0.0625, 0.125, and 0.25%) exposed to different time intervals (10 min to 24 h)

To assess the effectiveness of chitosan-capped AuNP as THI, the frozen AuNPs prepared from different concentrations of chitosan were exposed to temperature abuse condition (37 °C) from 0 to 48 h and its UV−Visible spectra and visible color were evaluated. As mentioned in the previous section, *λ*_max_ was observed at 530 and 540 nm for 0.125% and 0.25% chitosan, respectively, and for lower chitosan concentration, a prominent peak was not observed (Fig. 1a). Upon freezing, peak intensity reduced considerably for AuNPs prepared with 0.125% chitosan (Fig. 1c). Visible color of AuNPs prepared from higher chitosan concentration (0.25%) maintained its cherry red color even after freezing, whereas AuNPs prepared from 0.125% chitosan concentration lost its bright cherry red color and a grayish color was formed (Fig. 1c). This resulted in feeble peak intensity when observed spectrophotometrically. Lowest chitosan concentration (0.0625%) did not produce any cherry red-colored AuNPs, but it remained as a light yellow color both before and after freezing. This could be attributed to the higher zeta potential of AuNPs prepared using 0.125 and 0.0625% chitosan, in which due to higher surface energy, particles got agglomerated and became unstable. Upon exposure to temperature abuse conditions, the peak intensity increased with increase in the period of temperature abuse for AuNPs capped with 0.125 and 0.25% chitosan concentrations (Fig. 3). However, there was no peak observed for the lowest concentration (0.0625%) of chitosan-capped AuNPs up to 24-h abuse period. Although chitosan concentration of 0.125% exhibited a peak, its intensity was very less compared to 0.25% chitosan. Chitosan-capped gold nanoparticles prepared from 0.25% concentration exhibited a clear temperature abuse indicator. UV–vis spectroscopy indicated a shift in the peak to the left with increase in temperature abuse duration (Fig. 3). The initial *λ*_max_ observed at 540 nm shifted to 520−524 nm (blue shift), which could be due to the aggregation of AuNPs upon exposure to temperature-abused conditions. Normally, unaggregated AuNPs will have a red color in solution and turn to blue to purple upon aggregation. Exposure to abused temperature below 4 h did not show any variations in the peak intensity, which showed a clear difference above 4 h. This indicates that chitosan-capped gold nanoparticles exhibit noticeable differences in the peak intensity when temperature abuse conditions are experienced for ≥4 h under frozen storage. Visible color of chitosan-capped gold nanoparticles also supports the findings of UV−Visible spectra. Only a higher concentration (0.25%) is effective to be used as an indicator for temperature abuse conditions or THI. Upon exposure from frozen storage to abused temperature, the bright cherry red color began to change with duration of abused temperature. It started to turn purple to dark grayish from 4 h onward, indicating a clear distinguishing visible color upon temperature abuse of ≥4 h. With further increase in temperature abuse period, it turned into a more intense gray color. This could be due to the aggregation of AuNPs and alteration in shape and size upon exposure to time and temperature abuse. Capping with chitosan also alters the absorption and scattering behavior of gold nanoparticles favorable to use as a temperature threshold sensor. In this study, chitosan was used as a reducing and stabilizing agent for the synthesis of gold nanoparticles. During the process, chitosan could quickly adsorb onto the surface of AuNPs, as indicated by FTIR results. As chitosan has a –NH_3_^+^ group, the chitosan-capped AuNPs are positively charged, which stabilizes AuNPs preventing aggregation even during frozen storage, particularly in 0.25% chitosan due to a large number of the –NH_3_^+^ group. When these chitosan-capped AuNPs are exposed to temperature fluctuations, it induces aggregation of chitosan-capped AuNPs leading to alteration in the visible color from cherry red to gray. This property was used to design a sensor for temperature abuse indicator. The chitosan-capped AuNP used for indicating the temperature abuse conditions during frozen storage is very simple to produce and easy to maintain. It provides reliable irreversible color changes with the temperature abuse and the temperature abuse indicator proposed in this study is disposable type. Very limited quantity (250–300 µL) will be sufficient to detect the changes in the chitosan-capped AuNP solution. The developed indicator can be placed onto the pack as labels without coming in contact with the product. However, further study is needed to investigate the mechanism involved in indicating visible color changes of chitosan-capped AuNP during temperature-abused conditions.

## Conclusion

Chitosan, a biopolymer of crustacean waste can be used as a reducing agent for the synthesis of gold nanoparticles, which is a greener method. Variation in the concentration of chitosan results in varying the size of AuNPs and exposure to temperature- abused condition resulted in a distinctly different color indicating its efficiency to use as THIs. This can be used to ensure the quality and safety of frozen stored perishable food and pharma products during shipment and transportation to distant locations. The preparation method is very simple and ecofriendly and easily adoptable by the industry.

## Materials and methods

### Materials

Low-molecular-weight chitosan (∼68,000 Da) procured from Sigma Aldrich Chemicals, USA was used in the study. Hydrogen tetrachloroaurate (III) trihydrate (HAuCl_4_^.^3H_2_O) was from Acros Chemicals (New Jersey, USA), whereas glacial acetic acid for the preparation of chitosan solution was procured from Fisher Scientific, USA.

### Methods

#### Properties of chitosan

Chitosan used in the study was analyzed for sensory appearance to assess its color. Moisture content was determined by drying a known amount of sample to constant weight in an air oven at 105 ± 2 °C for 16 h.^23^ The percentage of crude protein was determined by the total nitrogen method.^23^ Ash content was determined by heating at 550 ± 2 °C in a muffle furnace.^23^ The degree of deacetylation of the chitosan was determined following the method of Shigemassa et al.^24^ The pH measurements of the chitosan solutions (1% w/v, in acetic acid) were carried out using a glass electrode digital pH meter (Cyberscan 510, Eutech Instruments, Singapore).^25^ Viscosity of chitosan solutions was determined at room temperature by using a digital viscometer (Brookfield DV-E Viscometer, Middleboro, MA) and values were expressed as centipoise (cP).

#### Optimization of conditions for synthesis of chitosan–AuNP and its characterization

Chitosan concentration (0.0625, 0.125, and 0.25%), heating temperature (70, 80, and 90 °C), and heating duration (5, 10, and 15 min) were used for optimization of synthesizing chitosan-capped AuNPs. For this, different concentrations of chitosan dissolved in 1% acetic acid were exposed to 90 °C maintained in a stirring water bath (Major Science, USA) for 30 min to dissolve the chitosan completely. To this, 5 mL of 10 mM HAuCl_4_^.^3H_2_O was added with continuous stirring and continued heating in the water bath maintaining different temperatures (70, 80, and 90 °C). The color evolution was observed for all the samples for different time intervals (5, 10, and 15 min). After this time interval, the samples were removed from the water bath and cooled immediately to room temperature using running water. Upon cooling, spectroscopic absorption (400–700 nm) of chitosan-capped AuNPs was measured using a UV−Visible spectrophotometer (Lambda 25, Perkin Elmer, USA). The colloidal chitosan-capped gold nanoparticles were directly used without any dispersing agents/diluents. The effect of chitosan concentrations on the size of the AuNPs, zeta potential, and polydispersity were assessed using a particle size analyzer (90 Plus Particle Size Analyser, Brookhaven Instruments Corporation, USA). In this, colloidal chitosan-capped gold nanoparticles were directly used without any dispersing agents. Disposable plastic cells are used for the analysis. Around 1.5 mL of chitosan-capped gold nanoparticles solution was added to the measuring cell and PTFE stoppers were used. Size distribution was measured by the dynamic light-scattering method using a particle size analyzer equipped with a Peltier temperature control system. Measurements were conducted at a fixed angle of 90° and a wavelength of 659 nm. Five replicate measurements were performed for each experimental condition. Each measurement consisted of five subsequent individual runs of 30 s duration. Fourier transform-infrared spectroscopy of pure chitosan and chitosan-capped AuNPs was performed using attenuated total reflectance-Fourier transform-infrared (ATR-FTIR) spectrophotometer (Spectrum 100, Perkin Elmer, USA). The stability of the AuNPs prepared using different concentrations of chitosan was assessed in the frozen storage conditions. For this, the frozen stored cuvettes containing chitosan-capped AuNPs were removed at regular intervals (0, 7, 15, 21, and 29 days) and thawed in the water bath (Thermomix 1420, USA) maintained at 37 °C and used for assessing the stability in terms of its size. After selecting the suitable concentration of chitosan initially, the concentration of chitosan was further optimized by varying chitosan concentration by ±0.05%.

#### Evaluation of chitosan-capped AuNPs as an indicator for monitoring frozen temperature abuse condition

Chitosan-capped gold nanoparticles were assessed for their temperature threshold sensing properties. For this, different chitosan concentrations (0.0625, 0.125, and 0.25% w/v), time (15 min), and temperature (90 °C) were used to prepare chitosan-capped AuNPs. The chitosan-capped gold nanoparticles colloidal solution was poured into 1.5-mL semi-micro disposable cuvettes (PMMA, Brandtech Scientific Inc., USA) and kept at a temperature of −18 °C for 6–7 days to freeze the solution. To simulate temperature abuse for frozen stored products, the cuvettes containing colloidal gold nanoparticles solution were exposed to higher temperature (∼37 °C) for varying duration (10 min to 48 h). After this, the cuvettes containing gold nanoparticles solution were replaced back to a frozen store and allowed to stabilize for 4 days. The variations in the visible color as well as UV−Visible spectrophotometer spectra upon exposure to temperature abuse were recorded and compared with the initial frozen sample. The visible color variations obtained for gold nanoparticles upon temperature abuse indicating its application for temperature threshold sensor were evaluated.

### Statistical analysis

The results are expressed as mean ± standard deviation. Experimental data were analyzed using the software SPSS version 10.00.^26^ For data analysis, mean, standard deviation, and analysis of variance were used. Significance of differences was defined at *P* < 0.05.

## Data Availability

The authors declare that the data supporting the findings of this study are available with the authors.

## References

[1] Singh, R. P. D. in *Shelf Life Evaluation of Food* 2nd edn (eds Man, C. M. D. & Jones, A.), 3−22 (Aspen Publishers, Gaithersburg, MD, 2000).

[2] Bagci PO, Wang YC, Gunasekaran S (2015). A simple and green route for room-temperature synthesis of gold nanoparticles and selective colorimetric detection of cysteine. J. Food Sci..

[3] Mohanpuria P, Rana NK, Yadav SK (2008). Biosynthesis of nanoparticles: technological concepts and future applications. Nanopart. Res..

[4] Mohan CO, Ravishankar CN, Lalitha KV, SrinivasaGopal TK (2012). Effect of chitosan edible coating on the quality of double filleted Indian oil sardine (*Sardinella longiceps*) during chilled storage. Food Hydrocoll..

[5] Ng CH, Hein S, Ogawa K, Chandrkrachang S, Stevens WF (2007). Distribution of D-glucosamine moieties in heterogeneously deacetylated cuttlefish chitin. Carbohyd Polym..

[6] Remya S (2016). Effect of chitosan based active packaging film on the keeping quality of chilled stored barracuda fish. J. Food Sci. Technol..

[7] Remya S, Mohan CO, Venkateswarlu G, Sivaraman GK, Ravishankar CN (2017). Combined effect of O_2_ scavenger and antimicrobial film on shelf life of fresh cobia (*Rachycentron canadum*) fish steaks stored at 2 °C. Food Cont..

[8] Renuka V, Mohan CO, Kriplani Y, Sivaraman GK, Ravishankar CN (2016). Effect of chitosan edible coating on the microbial quality of Ribbonfish, Lepturacanthussavala (Cuvier, 1929) steaks. Fish. Technol..

[9] Huang H, Yang X (2004). Synthesis of chitosan-stabilized gold nanoparticles in the absence/presence of tripolyphosphate. Biomacromolecules.

[10] Wang B (2006). Chitosan-mediated synthesis of gold nanoparticles on patterned poly (dimethylsiloxane) surfaces. Biomacromolecules.

[11] Chen Z, Wang Z, Chen X, Xu H, Liu J (2013). Chitosan-capped gold nanoparticles for selective and colorimetric sensing of heparin. J. Nanopart. Res..

[12] Yang N, Li W (2015). Preparation of gold nanoparticles using chitosan oligosaccharide as a reducing and capping reagent and their in-vitro cytotoxic effect on human fibroblast cells. Mater. Lett..

[13] Boyles MSP (2015). Chitosan functionalization of gold nanoparticles encourages particle uptake and induces cytotoxicity and pro-inflammatory conditions in phagocytic cells, as well as enhancing particle interactions with serum components. J. Nanobiotechnol..

[14] Wang YC, Lin L, Gunasekaran S (2017). Biopolymer/gold nanoparticles composite plasmonic thermal history indicator to monitor quality and safety of perishable bioproducts. Biosens. Bioelectron..

[15] Wang YC, Lin L, Gunasekaran S (2015). Gold nanoparticle-based thermal history indicator for monitoring low-temperature storage. Microchim. Acta.

[16] Li QD, Dunn ET, Grandmaison EW, Goosen MFA (1992). Applications and properties of chitosan. J. Bioact. Compat. Pol..

[17] Wang YC, Gunasekaran S (2012). Spectroscopic and microscopic investigation of gold nanoparticle nucleation and growth mechanisms using gelatin as a stabilizer. J. Nanopart. Res..

[18] Adlim A, Bakar MA (2008). Preparation of chitosan-gold nanoparticles: Part 2. The role of chitosan. Indones. J. Chem..

[19] Marchessault, R. H., Ravenelle, F. & Zhu, X. X. *Polysaccharides for Drug Delivery and Pharmaceutical Applications* (American Chemical Society, New York, 2006).

[20] Mano JF, Koniarova D, Reis RL (2003). Thermal properties of thermoplastic starch/synthetic polymer blends with potential biomedical applicability. J. Mat. Sci. Mat. Med..

[21] Nunthanid J, Puttipipatkhachorn S, Yamamoto K, Peck GE (2001). Physical properties and molecular behavior of chitosan films. Drug Dev. Ind. Pharm..

[22] Xu Y, Kim K, Hanna M, Nag D (2005). Chitosan-starch composite film: preparation and characterization. Ind. Crop. Prod..

[23] AOAC. *Official Methods of Analysis*, 17th edn, Vol. II, 1−27 (Maryland, USA, 2000).

[24] Shigemassa Y, Matsuura H, Sashiwa H (1996). Evaluation of different absorbance ratios from infrared spectroscopy for analyzing the degree of deacetylation in chitin. Int. J. Biol. Macromol..

[25] IS: 2168. *Specification for Pomfret Canned in Oil* (Indian Standard Institute, New Delhi, India, 1971).

[26] SPSS. *Statistical Software Package for Social Sciences for Windows—Release 10* (SPSS, Chicago, IL, 2000).

